# IP6K gene identification in plant genomes by tag searching

**DOI:** 10.1186/1753-6561-5-S2-S1

**Published:** 2011-05-28

**Authors:** Fabio Fassetti, Ofelia Leone, Luigi Palopoli, Simona E Rombo, Adolfo Saiardi

**Affiliations:** 1 DEIS, Università della Calabria, Via Pietro Bucci 41C Rende (CS) Italy; 2 LMCB, MRC Cell Biology Unit & Department of Developmental Biology, University College London, Gower Street, London, UK

## Abstract

**Background:**

Plants have played a special role in inositol polyphosphate (IP) research since in plant seeds was discovered the first IP, the fully phosphorylated inositol ring of phytic acid (IP6). It is now known that phytic acid is further metabolized by the IP6 Kinases (IP6Ks) to generate IP containing pyro-phosphate moiety. The IP6K are evolutionary conserved enzymes identified in several mammalian, fungi and amoebae species. Although IP6K has not yet been identified in plant chromosomes, there are many clues suggesting its presences in vegetal cells.

**Results:**

In this paper we propose a new approach to search for the plant *IP6K* gene, that lead to the identification in plant genome of a nucleotide sequence corresponding to a specific tag of the *IP6K* family. Such a tag has been found in all *IP6K* genes identified up to now, as well as in all genes belonging to the Inositol Polyphosphate Kinases superfamily (*IPK*). The tag sequence corresponds to the inositol-binding site of the enzyme, and it can be considered as characterizing all *IPK* genes. To this aim we applied a technique based on motif discovery. We exploited DLSME, a software recently proposed, which allows for the motif structure to be only partially specified by the user. First we applied the new method on mitochondrial DNA (mtDNA) of plants, where such a gene could have been nested, possibly encrypted and hidden by virtue of the editing and/or trans-splicing processes. Then we looked for the gene in nuclear genome of two model plants, *Arabidopsis thaliana* and *Oryza sativa*.

**Conclusions:**

The analysis we conducted in plant mitochondria provided the negative, though we argue relevant, result that *IP6K* does not actually occur in vegetable mtDNA. Very interestingly, the tag search in nuclear genomes lead us to identify a promising sequence in chromosome 5 of *Oryza sativa*. Further analyses are in course to confirm that this sequence actually corresponds to *IP6K* mammalian gene.

## Background

Plants have played a special role in inositol polyphosphates research ever since the first inositol polyphosphate (inositol hexakisphosphate) was described about 90 years ago in plant seeds [1]. Interest in inositol polyphosphates dramatically increased about thirty years ago when the role of inositol 1,4,5-trisphosphate (Ins(1,4,5)P_3_) in mobilization of Ca^2+^ from intracellular stores was discovered [2]. It is now clear that inositol polyphosphates are an important class of signaling molecules controlling disparate cellular functions. Inositol hexakisphosphate (IP6, also known as phytic acid) is the most abundant inositol polyphosphate in eukaryotic cells. It is a major component of plant seeds representing 0,1 – 1% of its dry weight and 60 – 80% of total phosphate content [3].

Significantly, IP6 is the precursor of a novel class of more anionic inositol polyphosphates, the inositol pyrophosphates, in which the fully phosphorylated IP6 ring is further phosphorylated to create high-energy pyrophosphate groups. The best charateryzed inositol pyrophosphates are the diphosphoinositol pentakisphosphate (IP7 or PP-IP5) and the bis-diphosphoinositol tetrakisphosphate (IP8 or [PP]2-IP4), with one and two pyrophosphate group, respectively [4].

Inositol pyrophosphates are important cellular messengers that control a wide range of cellular function, including endocytosis [5], apoptosis [6], telomere length [7], DNA recombination [8]. The high energy pyrophosphate bond of IP7 can directly donate the beta phosphate to proteins defining a new kind of protein phosphorylation mechanism [9], recently proposed to represent a novel post transductional protein modification (serine pyro-phosphorylation) [10].

Since their discovery in the early 1990s, inositol pyrophosphates have been found in all eukaryotic cells analyzed, from yeast to mammalian neuron, along with the widespread conservation of the enzymes responsible for their synthesis.

The mammalian enzymes responsible for IP7 synthesis are called IP6 Kinases (IP6Ks); they are able to convert IP6 plus ATP to IP7 and ADP [11]. It is now known that IP6Ks belong to a superfamily of Inositol Polyphosphate Kinases (PFAM accession number PF03770), that evolved from a common ancestor, comprising IP6Ks, Inositol Polyphosphate Multikinase (IPMK) possessing a broad range of substrates and IP3-3Ks that specifically convert I(1,4,5)P3 to I(1,3,4,5)P4. Although IP6K has not yet been identified in plant genomes, the presence of pyrophosphate IP7 has been demonstrated also in vegetal organisms, both in monocotyledonous and in dicotyledonous plants [12,13]. Furthermore, the conversion of IP6 to IP7 has been detected in *Arabidopsis* cells and leaf tissue in the presence of ATP, demonstrating IP6-kinase activity in plant extracts [14]. These findings, together with the observed high conservation through the evolution of IP6K, strongly suggest the presence of this enzyme in vegetal cells. Therefore, IP6K enzyme was searched in plant genomes by homology based methods, but all studies have failed to reveal its presence. Two IPMK proteins (called AtIPK2a and AtIPK2b in *Arabidopsis thaliana*) have been identified so far [15,16]. These two enzymes contribute to inositol 1,3,4,5,6-pentakisphosphate(IP5) production in *Arabidopsis*, but do not show any inositol pyrophosphate enzymatic activity [15,16]. In rice and barley an IPMK able to phosphorylate all intermediates from inositol bisphosphate to IP6 has been characterized [17]. To date IPMKs have been identified in dicotyledonous and in monocotyledonous plants, as well as in algae. There are many clues connecting IP6K to cell mitochondria. It was shown that human IP6K2 moves from nuclei to mitochondria and provides physiologic regulation of apoptotic process by generating IP7 [18]. Furthermore, yeast deficient in KCS1 (yeast IP6-Kinase), *kcsl*∆, do not survive if they are grown in conditions in which survival is dependent from mitochondrial function, thus demonstrating the importance of IP6K for this organelles [19]. Summarizing, to date *IP6K* has not been identified in plant chromosomes, but there are many clues suggesting its presence in vegetal cells. Some further observations could suggest that the corresponding gene might be found in plant mtDNA, probably encripted and hidden by virtue of editing and/or trans splicing processes. It is known that most of mtDNA information concerns genic products acting inside the mitochondrion itself. Plant mitochondrial genomes have several peculiar characteristics such as the large size (from 200*Kb* to 2400*Kb*), the presence of introns and genetic material of chloroplast or nuclear origin [20].

Furthermore, mitochondrial genome is characterized by occurrence of RNA editing and trans splicing mechanism enlarging protein variability [21]. RNA editing is a process in which some bases of an RNA molecule are enzymatically modified, so that its information content can be altered. Many molecular editing mechanisms are known, but in plants the most frequent is cytidine to uridine transformation. In plant mitochondria RNA editing is very common and it is required for gene expression. Actually the genomic information encoding an open reading frame is often incomplete in these organelles, and RNA editing is necessary to yield a functional product. The amino acid sequence of the encoded protein is effectively altered after editing process, so that it differs from that predicted by the genomic DNA sequence. Trans splicing is a further process generating genetic variability, in which two RNA molecules, produced by different DNA regions (even very distant from one another), are joined in a single RNA molecule able to produce a protein.

On the basis of the above considerations, we decided to search IP6K gene in mtDNA of plants as well as in nuclear chromosomes of two model vegetal organisms. Thus, we analyzed all published mtDNA of plant and the whole nuclear genome of *Arabidopsis thaliana* and *Oryza sativa*, a dicotyledonous and a monocotyledonous plant respectively. *Arabidopsis thaliana* is a small flowering plant, belonging to eudicot, the largest group of flowering plants on the planet. Because of its short generation time and compact size, it is used as a model organism in plant biology and genetics. Its nuclear genome comprises five chromosomes, with a total size of approximately 125 Mb (megabases). It is one of the smallest genome among plants, and it was the first plant genome to be sequenced in 2000 [22]. *Oryza sativa* (rice) was the second plant genome to be published [23], the first among monocot. It has the smallest cereal genome consisting of just 430 Mb across 12 chromosomes and it is routinely used as a model organism in cereal genomics.

Because of the considerable sequence heterogeneity among the several known IPKs, common homology search programs are not useful to our aim. Thus, we decided to use a new approach, looking not for the gene sequence as whole, but for a specific tag sequence, characterizing *IPK* gene family. This is possible only when a gene, or a gene family, contains a region (usually a short sequence) that is indispensable and always present in the gene sequence. In fact, alignment studies between IPKs from different organisms allowed to identify several conserved motifs in the amino acids sequence. These motifs comprise the ATP binding site, first characterized in IP3-3K [24], the C-terminal motif (last 19 amino acids), important for the catalytic activity [25], the “SSLL” motif, required for enzymatic activity of IP6K [26] and the P-XXX-D-X-K-X-G tag, a sequence of nine amino acids with four of them very conserved among IPKs [11]. Despite the considerable sequence heterogeneity of IPKs, this last motif represents a unique consensus sequence and it can be considered a specific tag of IPK gene family. The consensus sequence P-XXX-D-X-K-X-G is a very important functional region, identifying the inositol binding site of the enzyme [27]. Here, the functional role explains its strong conservation through evolution. To search for the IPK family tag in plant DNA we exploited DLSME [28], a software for motif extraction which allows for the motif structure to be only partially specified by the user, as better explained in the following.

## Methods

Common softwares for sequence search are based on sequence similarity, but they are not very useful when the expected homology between the gene searched for and the known sequences is low. Furthermore, these softwares cannot detect possible changes in nucleotide sequences due to RNA editing mechanisms. The intuition behind our work is that some specific gene families, such as all *IPK* genes, are characterized by the presence of specific tags, short sequences of few amino acids, often corresponding to functional regions. Thus, we present a general, semi-automatic methodology to discover the possible presence of specific, still undiscovered, genes in cells and we applied it to plant genomes.

Such a methodology can be summarized as follows:

1. (*Tag Definition*) set a (partially undefined) sequence representing the specific tag to search for;

2. (*Genome Scanning*) scan a plant genome sequence (or a set of genome sequences) to individuate possible instances of the tag;

3. (*Post-processing Analysis*) analyze the candidate subsequences extracted by the previous step in order to verify the presence of the gene in the considered genomes.

In particular, the sequence associated to the tag defined by step 1 is made of both symbols in the alphabet Σ = {*A,C,G,T*}, representing nucleic acids, and a generic symbol *X* that can be associated to a subset of Σ. This way, step 2 can be carried out by performing an approximate search of the *motif* represented by the tag sequence.

In the following we specify how the steps listed above have been particularized to achieve our purposes.

### Tag Definition

The most important tag for IPK gene is the P-XXX-D-X-K-X-G motif, corresponding to the inositol binding site of the enzyme. Thus, for the identification of *IP6K* gene in plant DNA, we focused on the nucleotide sequence corresponding to this specific IPK tag.

### Genome Scanning

We analyzed all the published mtDNAsequences (available at http://www.ncbi.nlm.nih.gov/sites/entrez) and the whole nuclear genome of two plants and performed motif extraction from them, since a tag can be viewed as a subsequence whose structure is not completely specified a priori. Among the different algorithms and tools available for motif discovery (e.g., see [29-34]), we chose DLSME [28] since it is able to handle different complex kinds of pattern variabilities, as will be better recalled in the following.

### Post-processing Analysis

For each identified tag, we extracted a sequence of about 1200 nucleotides surrounding the consensus sequence and examined it as a candidate IP6K gene. Nucleotidic sequences were translated in aminoacid sequences by using the Transeq [35] software. Then, we examined the identified amino acid sequences looking for other IP6Ks conserved domains. In order to detect possible homologies, we performed sequence alignments using ClustalW [36] and BLAST [37]. Finally, using the TBLASTX and TBALSTN algorithms, we screened expressed sequence tag (EST) databases for proteins containing the sequences identified by our tag search.

In the following, we first provide a brief description of DLSME and of the setting we exploited for our purposes, and then describe the main results we have obtained by our analysis.

### Using DLSME

DLSME [28] is a system designed to mine general kinds of motifs where several “exceptions” may be tolerated; that is, it is able to handle different complex kinds of pattern variabilities. In particular, DLSME is able to search for patterns composed of any number of short subsequences (boxes, in the following), where the sizes of both the conserved regions and the regions between two boxes can be specified by the user as intervals ranging from a minimum to a maximum value. Moreover, mismatches are taken into account, as well as “skips” (deletions) and box “swaps” (box invertions), that possibly affect box occurrences. Furthermore, in DLSME, it is possible to specify boxes where some symbols are “anchored” to get a fixed value. Despite the complexity of the allowed pattern variabilities, the system is able to exhibit good performances.

**Figure 1:**
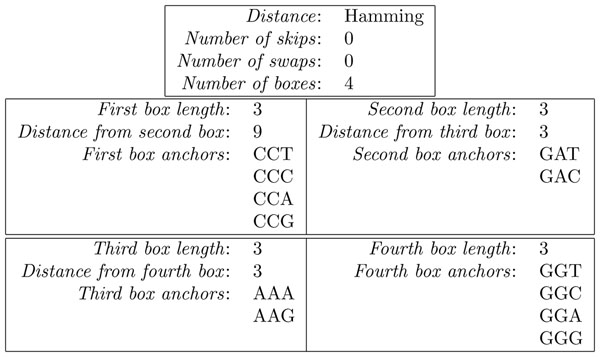
**DLSME configuration parameters** Configuration parameters exploited for DLSME when we looked for the pattern: CC{T,C,A,G} -------- GA{T,C}---AA{A,G}---GG{T,C,A,G}

In particular, for the purposes of this research, we looked for the pattern:

CC{T,C,A,G} -------- GA{T,C}---AA{A,G}---GG{T,C,A,G}

using the sets of DLSME configuration parameters reported in Figure 1.

## Results

**Figure 2:**
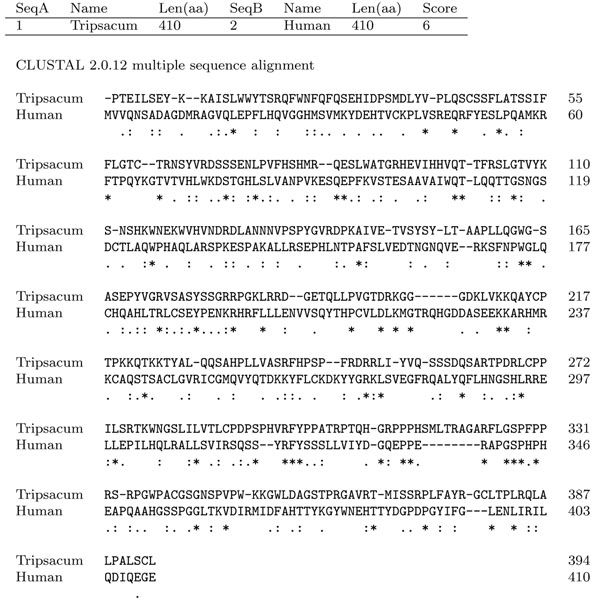
**Alignment around PVGTDRKGG vs human** Alignment between the 410 amino acids around the PVGTDRKGG sequence of Tripsacum dactyloides and the human IP6K gene (ClustalW2). “*” = residues identical in the two sequences in the alignment; “:” = conserved substitutions; “.” = semi-conserved substitutions.

Due to the numerous suggestions relating IP6K to cell mitochondria, we decided to first perform the *IP6K* gene search on mitochondrial DNA of plants. To date the full mitochondrial genome sequence is known for 42 different vegetal organisms, belonging to various Phyla, even very distant from one another from the evolutionary point of view. The specific IP6Ks tag (P-XXX-D-X-K-X-G) was searched over the overall sequenced mitochondrial genomes available to date and both DNA strands were analyzed. Twenty three genomes out of 42 gave at least one positive match. Interestingly, we noted that some tag sequences (9 amino acids) were identical among different organisms. For each identified tag we extracted a sequence of about 1200 nucleotides surrounding it. To find out possible relevant homologies, we performed alignments among the sequences found in different vegetal organism. All the sequences sharing the same tag showed high similarity in the region surrounding the consensus sequence, while alignment with *IP6K* known genes (Saccharomyces cerevisiae *KCS1* or human *IP6K1*) showed only a weak similarity. Furthermore, in order to confirm the identity of our putative hit, we looked for other *IP6Ks* conserved motifs in the identified putative amino acids sequence like the ATP binding site, the C-terminal motif (last 19 amino acids), and the “SSLL” motif. These analyzes led us to focus on the sequence PVGTDRKGG, that was found in mitochondrial genome of Tripsacum dactyloides, Sorghum bicolour, and three different species of Zea genus (Zea mays, Zea perennis and Zea luxurians). Alignment between the 410 aminoacid around the PVGTDRKGG sequence of Tripsacum dactyloides and the human *IP6K* gene showed an interesting correspondence of the consensus region (see Figure 2).

**Figure 3:**
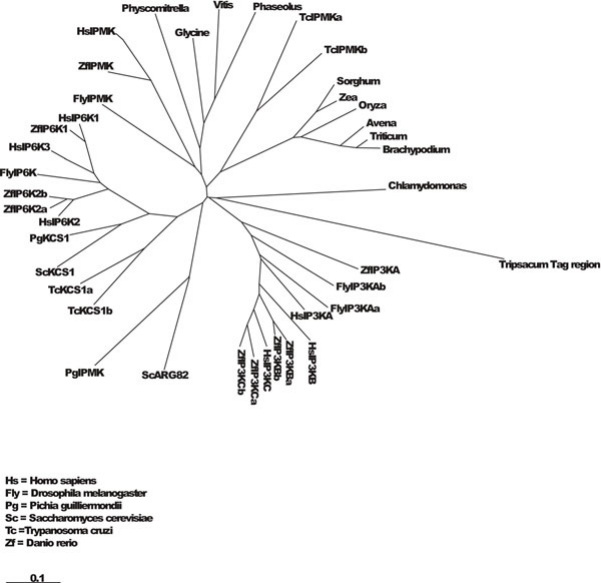
**Multiple alignment by ClustalW2** Phylogenetic tree from multiple alignment of a 50 amino acid region of Tripsacum dactyloides mtDNA surrounding the tag with corresponding regions of inositol phosphate kinase (IPMK, IP6K, IP3-3K) from different organism (Clustal W2). Branch lengths are proportional to the amount of inferred evolutionary change.

To verify if the Tripsacum dactyloides sequence was an actively transcribed gene, we analyzed the Expressed Sequence Tags (ESTs) databases. These databases include short fragments of DNA derived from a longer cDNA sequence and representing part of the expressed genome. In order to confirm the expression of our mtDNA sequence, we screened EST databases using the region surrounding the PVGTDRKGG tag of Tripsacum dactyloides. This search failed to find any EST matching indicating that our putative hit is unlikely to be transcribed in mRNA. Finally, we used a region of 50 amino acids of Tripsacum dactyloides mtDNA surrounding the consensus sequence to perform a multiple alignment with corresponding regions of inositol phosphate kinases (IPMK, IP6K, IP3-3K) from different organism using ClustalW2. As shown in Figure 3, our sequence resulted to be an outsider. This result indicated that the identified mitochondrial tag does not belong to any subgroup of kinases composing the *IPK* gene family.

**Figure 4:**
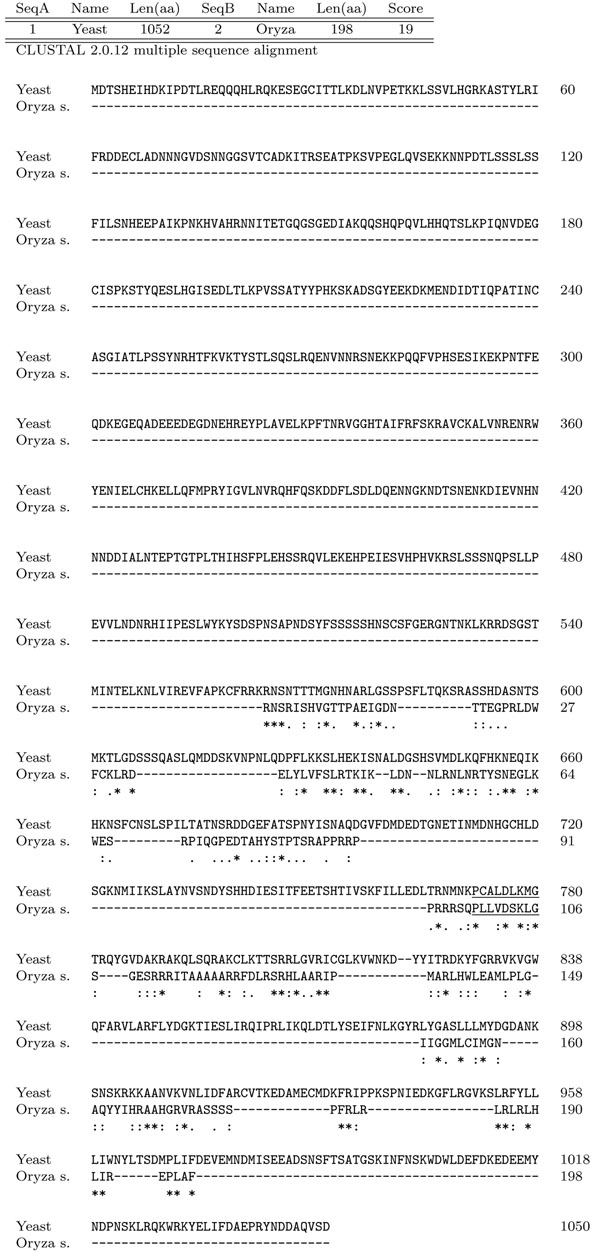
**Alignment around PLLVDSKLG vs yeast** Alignment between the 198 amino acid sequence around the PLLVDSKLG tag of *Oryza sativa* and the yeast KCS1 gene (Clustal W2). ”*” = residues identical in the two sequences in the alignment; ”:” = conserved substitutions; ”.” = semi-conserved substitutions. In red the P-XXX-D-X-K-X-G tag.

**Figure 5:**
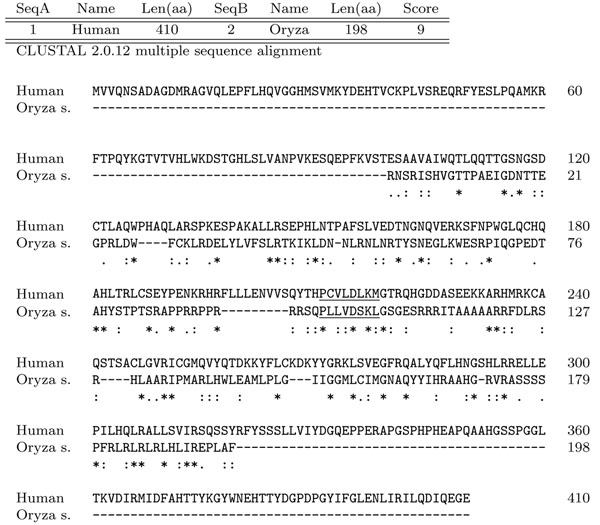
**Alignment around PLLVDSKLG vs human** Alignment between the 198 amino acid sequence around the PLLVDSKLG tag of *Oryza sativa* and the human IP6K gene (Clustal W2). ”*” = residues identical in the two sequences in the alignment; ”:” = conserved substitutions; ”.” = semi-conserved substitutions. In red the P-XXX-D-X-K-X-G tag.

**Figure 6:**
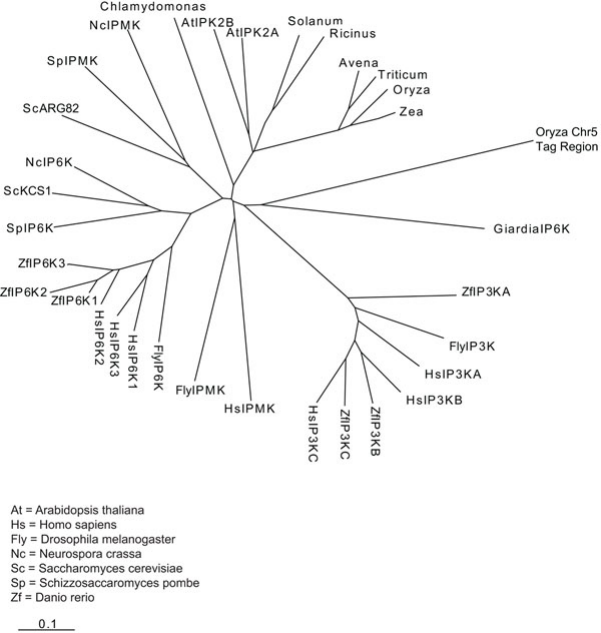
**Phylogenetic tree** A Phylogenetic tree from multiple alignment of a 50 amino acid region of *Oryza sativa* DNA surrounding the tag with corresponding regions of inositol phosphate kinase (IPMK, IP6K, IP3-3K) from different organisms (ClustalW2). Branch lengths are proportional to the amount of inferred evolutionary change.

Once excluded the presence of *IP6K* gene in mtDNA, we decided to look in nuclear genome of plants where, up to now, the search has been performed only by methods based on sequence similarity. We analyzed all chromosomes of *Arabidopsis thaliana* and *Oryza sativa*, a dicotyledonous and a monocotyledon plant respectively. In each chromosome, we found dozens of tags, but only few tag sequences per chromosome resulted in good candidates to be specific IP6K tags. In fact, too polar or too big amino acids between the four fixed positions of the tag are not consistent with the tag sequence functionality. In particular we considered as good candidate a tag sequence including amino acids *L*,*V*,*T*,*M*,*I*,*A*,*S*,*G*,*C* between the four fixed positions, and we rejected others. For each identified tag, we extracted a sequence of about 400 amino acids surrounding the tag. Each sequence was examined as a candidate *IP6K* gene as described above for mtDNA. We did not find any strong homology with known IP6Ks. This result was not surprising, because only a weak similarity is anyhow expected between organisms very distant from an evolutionary point of view. Thus, the selected sequence to be actually interesting was established on the basis of other parameters, like alignment of tag sequences, presence of other conserved amino acids and of sequence in EST database. A very promising sequence was found on chromosome 5 of *Oryza sativa*, around the tag PLLVDSKLG. The sequence comprises 198 amino acids without any stop codon. As shown in Figure 4, the ClustalW alignment of this sequence and Saccharomyces cerevisiae *KCS1* gene gave a positive score with an alignment in correspondence of the inositol-binding region. Alignment with human *IP6K* (*hIP6K*) gave a lower score, but still maintained the correspondence between tags (see Figure 5). We performed a multiple alignment (ClustalW2) between the region of 50 amino acids of *Oryza sativa* surrounding the tag and the corresponding regions of inositol phosphate kinases (IPMK, IP6K, IP3-3K) from different organisms. This analysis revealed (Figure 6) that the *Oryza sativa* sequence, although appear dissociated from other IPK family members shows a certain degree of similarity with *Giardia lamblia* IP6K, that itself appears to be a distant member of the *IPK* genes family. Finally, we screened the EST databases using the region surrounding the PLLVDSKLG tag of *Oryza sativa*. This search showed some matching EST, indicating that the tag sequence is likely to be transcribed in mRNA.

## Discussion and Conclusions

Inositol hexaphosphate kinase (IP6K) catalyzes the conversion of IP6 to IP7 using ATP as phosphate donor. It belongs to an inositol polyphosphate kinase superfamily, the IPKs (Pfam PF03770), that evolved from a common ancestor. It is thought that a primordial IPMK may have been the evolutionary precursor of the IP3-3Ks and the IP6Ks, all of which contain the P-XXX-D-X-K-X-G motif [38]. Moreover, this motif represents a unique consensus sequence for the IPK family, with four key amino acids very conserved among different inositol phosphate kinases, despite their considerable sequence heterogeneity. This region modulates the catalytic site for phosphate transfer from ATP to the inositol ring [39].

The inositol pyrophosphate IP7 is present in all eukaryotic cells analyzed thus far, from amoeba to man; it is not surprising that the enzyme responsible for its synthesis is highly conserved through evolution. In fact, after the first IP6K purification from rat brain [40], the enzyme was cloned in other mammalians, and its high evolutionary conservation was regularly observed, which facilitated the identification and cloning of IP6K enzymes from distant organisms, including yeast and the amoeba *Dictyostelium*[41]. It is notable that *Dictyostelium* diverted from the evolutionary main stream after the diversion of yeast but before the splitting between animals and plants [42]. Furthermore, the only *IPK* gene present in the ancient eukaryote diplomate *Giardia lamblia* has been demonstrated to be a *IP6K*[43]. Thus, on the basis of evolutionary considerations, *IP6K* is expected to be found also in vegetal organisms.

Moreover, pyrophosphate IP7 is present in vegetal organisms, and IP6-kinase enzymatic activity has been demonstrated in plants. However, bioinformatics analysis failed to identify any IP6 kinase in the complete *Arabidopsis thaliana* nuclear genome. We hypothesized that *IP6K* gene might actually occur nested in vegetal mtDNA, where more frequently phenomena enlarging protein variability do occur. Tag identification in mtDNA could indicate the presence of *IP6K* gene, even if not in a canonic form. Indeed, trans splicing mechanisms might compact a gene consisting of more segments dislocated in different mtDNA regions, and editing phenomena could contribute to the failure of homology searches. In fact, editing mechanism might generate RNA molecules much different from DNA producing them, so that DNA sequence can be not immediately referable to *IP6K* gene in its transcript. Thus, the search of a gene starting from its characterizing consensus sequence represents a promising approach to find an encrypted gene. We searched for a specific IP6K tag within all available vegetal mtDNA sequences using DLSME, a very flexible system for motif discovery, allowing for dealing with genetic code degeneration and possible occurrences of editing events. Our search revealed several tags in mtDNA of examined plants, but an accurate analysis of sequences surrounding the consensus motifs led us to conclude that our hit does not belong to the IPK gene family. Indeed, the P-XXX-D-X-K-X-G consensus sequence is a characterizing motif of IP kinases, and it was found in all members of the family. Our search failed to find any sequence containing the tag ascribable to *IP6K* gene and, thus, we can conclude that *IP6K* gene is not present in plant mtDNA.

Therefore, we decided to extend the search of *IP6K* gene on nuclear genome of plants. Up to now, *IP6K* gene search in plant chromosomes have been performed with bioinformatics methods based on sequence similarity. In our case, we looked at plant nuclear genome applying the new approach of gene identification by tag search. The advantage of this method is that it allows identification of a gene even if many nucleotidic changes have been accumulated during the evolution, so that the homology between homologous loci is now very low. In fact, it is known that *IP6K* is a gene highly conserved through mammalian evolution, but the homology is low when compared with organisms filogenetically very distant, like Yeast. It is possible that in evolutionary stream bringing to plants, many nucleotidic changes occurred, so that plant *IP6K* gene looks quite different both from mammalian and yeast genes. By carrying out our search, we found an interesting sequence in nuclear genome of *Oryza sativa*. This sequence shows an interesting similarity with yeast KCS1, giving a relatively high score when the two sequences are aligned using ClustalW. *KCS1* gene is quite different from mammalian *IP6K* genes. It is bigger, comprising 1052 amino acids against 410 of human IP6K, that lacks the first 305 KCS1 amino acids, and it has some other interruptions as compared to the yeast gene. Very interestingly, the homology region between *Oryza sativa* sequence and KCS1 is indeed clustered in the protein domain corresponding to human IP6K. This result might represent the strong evolution drive of the catalytic IPK domain and the likely conservation of the key feature of this domain in the identified *Oryza sativa* tag. Furthermore ClustalW alignment shows a correspondence between tags when we compare our sequence with both KCS1 and human or mouse IP6K. This correspondence is still maintained in multiple alignment between our sequence and KCS1, human IP6K and mouse IP6K.

Multiple alignment of a 50 amino acid region of *Oryza sativa* DNA surrounding the tag with corresponding regions of IPKs from different organisms showed a degree of connection between *Oryza sativa* tag and *Giardia lamblia* IP6K sequences. Interestingly, among the different inositol phosphate kinases tested, the best match of *Oryza sativa* tag region was with a very distant IP6K. This result suggested that the sequence around the identified tag might represent a distant member of the *IP6K* subfamily of gene as the *Giardia* IP6K enzyme.

As remarked above, the screening of EST databases showed some matching ESTs. Note that, although EST database are a very powerfull tools to study the trascriptome of a specific organism, they are often inperfect. Indeed, their quality is affected by transcript redundancy, low sequence quality and by high transcript truncation rates. Furthermore, these databases only represent the trascriptome of the tissue and developmental stages of the plant from which the mRNA was isolated. Thus, EST databases are not exhaustive, and a negative match does not exclude the expression of rare transcripts. This means that the ESTs we found indicate the chromosome region containing the putative plant IP6K is actively transcribed, although such ESTs do not possess the conserved PLLVDSKLG domain. Likely, the identified EST correspond to truncated isoform of the full length mRNA.

In conclusion, we think that this sequence is part of an *Oryza sativa* gene homologous to mammalian *IP6K*. In particular we suppose that it is the central part of the gene, comprising the inositol binding site, and it lacks in the N-terminus and the C-terminus sequences, thus indicating the presence of more than one exons in the rice gene. The big evolutive distance between rice and both human and yeast could explain the low similarity observed among these gene.

As future step of our research, we are planning experiments of molecular cloning and biochemical characterization to confirm our hypothesis and to determine substrate specificity of the enzyme. We will use RT-PCR to clone *Oryza sativa* IP6K cDNA. The cDNA will be cloned into a yeast expression vector and the activity will be assessed through trans-complementation of several yeast mutants, with particular focus on the yeast IP6 kinase mutant (*kcs*1∆). The recombinant enzymes will be tested *in vitro* by using either different [^3^H]inositol polyphosphates species or unlabelled inositol phosphates with [γ-^32^P]ATP to determine substrate specificity and calculate kinetic parameters.

## Competing interests

The authors declare that they have no competing interests.

## Authors’ contributions

The idea of applying tag searching to discover genes in plant genomes has been proposed by A. Saiardi. O. Leone defined the underlying methodology and carried out the experimental analysis, supported by F. Fassetti and S. E. Rombo for the computer science aspects of the matter. L. Palopoli and A. Saiardi coordinated the research. All five authors contributed equally to the writing of the manuscript.

## Supplementary Material

Additional file 1 Accession numbers of the genes referred in the figures. Click here for file
