# Supplementary material for: IP6K gene identification in plant genomes by tag searching
Source: BMC Proc. 2011 May 28;5(Suppl 2):S1. doi: 10.1186/1753-6561-5-S2-S1 (PMC3090757; doi:10.1186/1753-6561-5-S2-S1)
Supplement: Additional file 1 — Accession numbers of the genes referred in the figures. [file 1753-6561-5-S2-S1-S1.pdf]

Accession numbers of the genes referred in the figures

Homo sapiens IP6K1	NP_695005.1
Homo sapiens IP6K3	NP_473452.2
Homo sapiens IP6K2	NP_001005909.1
Homo sapiens IPMK	NP_689416.1
Homo sapiens IP3KA	NP_002211.1
Homo sapiens IP3KB	CAB65055.3
Homo sapiens IP3KC	NP_079470.1
Drosophila melanogaster mIP6K	NP_726095.2
Drosophila melanogaster IPMK	NP_608535.1
Drosophila melanogaster IP3K	NP_001096969.1
Saccharomyces cerevisiae IPMK	EDN60511.1
Saccharomyces cerevisiae IP6K	CAY78526.1
Schizzosaccaromyces pombe IPMK	NP_593593.1
Schizzosaccaromyces pombe IP6K	NP_587847.1
Giardlia lambia IP6k	XP_001706837.1
Danio rerio IPK1	XP_692904.2
Danio rerio IPK2	NP_958878.1
Danio rerio IPK3	NP_001002389.2
Danio rerio IPK4	NP_001073533.1
Danio rerio IP3KC	XP_002665435.1
Danio rerio IP3KB	XP_686458.2
Danio rerio IPK7	CAQ14679.1
Neurospora crassa IP6K	XP_956825.1
Neurospora crassa IPMK	XP_962577.2
Arabidopsis thaliana IPK2B	NP_200984.1
Arabidopsis thaliana IPK2A	NP_196354.1
Ricinus	XP_002511062.1
Solanum	ABO72543.1
Avena	GR329396.1 (PlantGDB)
Triticum	CK211413.1 (PlantGDB)
Zea	NP_001146961.1
Oryza	NP_001046986.1
Chlamydomona	XM_001694116.1
Sorgum	XP_002452184.1
Brachypodium	Bradi3g44560.1 (from the Brachypodium genome)
Phaseolus	CAP08583.1
Vitis	XP_002279587.1
Glycine	ABU93830.1
Physcomitrella	XP_001757782.1
Pichia guilliermondii IPMK	XP_001483490.1
Pichia guilliermondii KCS1	EDK38027.2
Trypanosoma cruzi KCS1a	XP_808099.1
Trypanosoma cruzi KCS1b	XP_814212.1
Trypanosoma cruzi IPMKa	XP_818026.1
